# Estimating COVID-19 cases in Makkah region of Saudi Arabia: Space-time ARIMA modeling

**DOI:** 10.1371/journal.pone.0250149

**Published:** 2021-04-20

**Authors:** Fuad A. Awwad, Moataz A. Mohamoud, Mohamed R. Abonazel

**Affiliations:** 1 Department of Quantitative Analysis, King Saud University, Riyadh, Saudi Arabia; 2 Department of Applied Statistics and Econometrics, Faculty of Graduate Studies for Statistical Research, Cairo University, Giza, Egypt; South China University of Technology, CHINA

## Abstract

The novel coronavirus COVID-19 is spreading across the globe. By 30 Sep 2020, the World Health Organization (WHO) announced that the number of cases worldwide had reached 34 million with more than one million deaths. The Kingdom of Saudi Arabia (KSA) registered the first case of COVID-19 on 2 Mar 2020. Since then, the number of infections has been increasing gradually on a daily basis. On 20 Sep 2020, the KSA reported 334,605 cases, with 319,154 recoveries and 4,768 deaths. The KSA has taken several measures to control the spread of COVID-19, especially during the Umrah and Hajj events of 1441, including stopping Umrah and performing this year’s Hajj in reduced numbers from within the Kingdom, and imposing a curfew on the cities of the Kingdom from 23 Mar to 28 May 2020. In this article, two statistical models were used to measure the impact of the curfew on the spread of COVID-19 in KSA. The two models are Autoregressive Integrated Moving Average (ARIMA) model and Spatial Time-Autoregressive Integrated Moving Average (STARIMA) model. We used the data obtained from 31 May to 11 October 2020 to assess the model of STARIMA for the COVID-19 confirmation cases in (Makkah, Jeddah, and Taif) in KSA. The results show that STARIMA models are more reliable in forecasting future epidemics of COVID-19 than ARIMA models. We demonstrated the preference of STARIMA models over ARIMA models during the period in which the curfew was lifted.

## Introduction

In univariate time series, we observe autocorrelation between the successive observations over a time frame. In building a time series model, the Autoregressive Moving Average (ARMA) models for spatial data can be viewed in a time series frame called space-time models. Space-time models explain the dependencies across space in the systematic dependency systems among the observations at various locations. Applications of spatial statistics cover many areas such that geostatistics, sociology, economics, and environmental.

Pfeifer and Deutsch [1] proposed the space-time autoregressive moving average (STARMA) model for some processes that can be modeled by STARMA. Models are characterized by a single random variable observed at N fixed points in space. The dependencies between the N time series are incorporated in the model through hierarchical *N*×*N* weighted matrices.

The STARMA model characterizes the spatial-temporal reliance between diverse districts by inferring the interaction between adjoining locales inside the system. So this model is more suitable for modeling geographic spatial-temporal data [1]; because this model completely considers the autocorrelation existing within the geographic spatial-temporal data set. In any case, the STARMA has certain restrictions in a few viewpoints, such as spatial and temporal non-stationary time series data modeling.

This paper is organized as follows: Section 2 reviews some literature about STARIMA Models, whereas Section 3 presents the used framework of time series data models. Section 4 introduces STARIMA models (STARMA, STAR, and STMA). Section 5 presents our empirical study on Makkah region states. Finally, Section 6 presents the conclusion and remarks.

## Literature review

The STARMA was first introduced by [2]. In 1979 Pfeifer started studying "Spatial-dynamic modeling" on his Ph. D. dissertation. Later, Deutsch joined him and became his co-author on "Stationarity and invertibility regions for low-order STARMA models". Pfeifer and Deutsch successively developed this model in the eighties and published articles as follows:

In 1980a, Pfeifer and Deutsch presented the three stages of building STARIMA models: identification, estimation, and diagnostic checking, and illustrated it with a substantive example. In the same year, Pfeifer and Deutsch [3] studied the characteristics of the STAR (1,1) and STMA (1,1) models as well as the behavior and patterns of the theoretical space-time autocorrelation, partial autocorrelation functions, and initial estimation. Pfeifer and Deutsch [4] also suggested a simulation of the experiment results to compare the accuracy of three alternative estimation techniques (maximum likelihood estimation, least squares estimation, and conditional maximum likelihood estimation) of STARMA model. Pfeifer and Deutsch [5] used the likelihood ratio test to develop an independence test of the STARIMA models’ residual.

Furthermore, Pfeifer and Deutsch [6] discussed the case of contemporaneously correlated variables in which the error covariance matrix (G) of the observations is non-spherical. In the same year, Pfeifer and Deutsch [7] presented Seasonal STARIMA models and discussed three stages for building the models. They illustrated the report with an example of their application. Pfeifer and Deutsch [8] also presented STARMA models where the covariance matrix of the innovations (*G* = *σ*^2^*I*) is not spherical. Also incorporated in these modeling procedures were tests of hypotheses concerning the form of G. The entire procedure was illustrated with two applications. Also, Pfeifer and Deutsch [9] developed approximate variances of the sample space-time autocorrelation function when the underlying process is white noise, which is needed to test the significance of the observed autocorrelations.

Recently, the STARMA models have been used in several studies, such as [10–12]. Moreover, Bayesian spatial and temporal approaches have been proposed in several studies, to take into account the effect of unobserved heterogeneity and spatio-temporal correlation, such as [13–16]

### Applied previous studies for COVID-19

Perone [17] used ARIMA model to forecast the epidemic trend for COVID-19 over the period after 4 April 2020, by using the Italian epidemiological data at national and regional levels. Results suggest that COVID-19 epidemic in Italy reach the plateau, in term of cumulative cases in May 2020 and the final epidemic size in Italy maybe around 200 thousand cases.

Tandon et al [18] developed a model for forecasting future COVID-19 cases in India. In their study, confirmed, recovered and death cases of COVID-19 infection were collected in India from 22 Jan 2020 to 13 April 2020. The study indicates an ascending trend for the cases in the coming days. The time series analysis also presents an exponential increase in the number of cases. The results for a measure of model accuracy for ARIMA, Linear Trend, Quadratic Linear, S-Curve Trend, Moving Average, Single Exponential and Double Exponential model suggests that ARIMA (2, 2, 2) model is the most accurate of all the models for forecasting future incidences as it possesses the least value for all the measures.

Iran was the first Middle East country to report a coronavirus case. Tran et al [19] developed a prediction model for COVID-19 in Iran. The model utilizes SARS-CoV-2 daily data, from 20 Feb to 4 May 2020, and ARIMA was employed to forecast the trend of the pandemic spread. The ARIMA model predicts that Iran can easily exhibit an increase in the daily total confirmed cases and the total deaths, while the daily total confirmed new cases, total new deaths, and growth rate in confirmed cases/deaths become stable in the near future. This study predicts that Iran can control the SARS-CoV-2 disease soon.

### COVID-19 in KSA

Alzahrani et al [20] employed ARIMA model to forecast the expected daily number of COVID-19 cases in Saudi Arabia. They used to perform four different prediction models of ARIMA models, and determine the best model ARIMA (2,1,1). The forecasting results showed that the trend in Saudi Arabia will continue growing and may reach up to 7668 new cases per day and over 127,129 cumulative daily cases by 21 May 2020, if stringent precautionary measures were not implemented to limit the spread of COVID-19. They pointed out that training the model on more data will lead to more reliable forecasting.

Elhassan and Gaafar [21] developed both the ARIMA model and Logistic growth model to study the trend and to provide short and long-term forecasting of the prevalence COVID-19 cases in Saudi Arabia. The data analyzed in their study covered the period of 2 Mar to 21 Jun 2020. ARIMA and Logistic growth models showed excellent performance in projecting the epidemic prevalence.

Among the effects of the pandemic is the suspension of the Hajj and Umrah events in the two holy cities of Mecca and Medina that were supposed to be performed by nearly two million Muslims in mid-July. Makkah is in the middle of the cities of Jeddah and Taif. Pilgrims and Umrah performers cross those cities come to Makkah to complete their religious journey. Noticeably, the spread of the COVID-19 in the Kingdom started from Mar 2020, as a result of the movement of pilgrims and residents between those cities. These movements led to the rapid spread of COVID-19 between the cities. Thus, this paper aims to study the spatial effects (neighborhood analysis) of infected cases of COVID-19 in Makkah, Jeddah, and Taif.

## ARIMA models

Box and Jenkins [22] is one of the most well-known traditional methods of time series modeling. It is also called Autoregressive Integrated Moving Average (ARIMA). Vandaele [23] explains that this method is divided into several models. It can be divided into stable and unstable models. The following is a brief presentation of the models for this method.

### Autoregressive (AR) models

These models are called Autoregressive models because the variable (*X*_*t*_) is linear with itself in previous time periods (*X*_*t*−1_, *X*_*t*−2_,…,*X*_*t*−*p*_). It is possible to express the current observation of the series (*X*_*t*_) as a linear function in its previous observations and random error limit, denoted by the symbol (*u*_*t*_). Autoregressive models can be formulated as follows:

$$X_{t}=\varphi_{1}X_{t-1}+\varphi_{2}X_{t-2}+\cdots +\varphi_{p}X_{t-p}+u_{t}$$ (1)

where *X*_*t*_ are observations of a stationary time series, *u*_*t*_ is a white noise process with mean zero and constant variance $\left(\sigma_{u}^{2}\right)$, and *φ*_1_, *φ*_2_,…,*φ*_*p*_ are autoregressive parameters of the model.

### Moving Average (MA) models

These models are called moving average models because the variable (*X*_*t*_) is linear with random errors limit in previous and current time periods (*u*_*t*_, *u*_*t*−1_, *u*_*t*−2_,…,*u*_*t*−*q*_). According to [24], Moving Average models can be formulated as follows:

$$X_{t}=u_{t}-\theta_{1}u_{t-1}-\theta_{2}u_{t-2}-\cdots -\theta_{q}u_{t-q}$$ (2)

where *θ*_1_, *θ*_2_,…,*θ*_*q*_ are moving average parameters of the model.

### Autoregressive Moving Average (ARMA) models

These models are called Autoregressive and Moving Average models because the variable *X*_*t*_ is linear with itself in previous time periods (*X*_*t*−1_, *X*_*t*−2_,…,*X*_*t*−*p*_), and the random errors limit in previous and current time periods (*u*_*t*_, *u*_*t*−1_, *u*_*t*−2_,…,*u*_*t*−*q*_). Autoregressive Moving Average models can be formulated as follows:

$$X_{t}=\varphi_{1}X_{t-1}+\cdots +\varphi_{p}X_{t-p}-\theta_{1}u_{t-1}-\ldots -\theta_{q}u_{t-q}+u_{t}$$ (3)

### Autoregressive Integrated Moving Average (ARIMA) models

Some time series models are non-stationary in mean, and thus uses differencing as for their transformation. A stationary differenced time series can be modeled with autoregressive moving average, but requires an inclusion of the differenced operator. Since the order of integration of a process is the number of differenced needed to achieve stationarity, we therefore name the model-autoregressive integrated moving average (ARIMA) model. The general form of this model is ARIMA (p, d, q):

$$W_{t}=\varphi_{1}W_{t-1}+\cdots +\varphi_{p}W_{t-p}-\theta_{1}u_{t-1}-\cdots -\theta_{q}u_{t-q}+u_{t}$$ (4)

where *W*_*t*_ is the first difference of *X*_*t*_, i.e., *W*_*t*_ = *X*_*t*_−*X*_*t*−1_.

## Framework of spatial time series models

After the emergence of STARIMA models, many theoretical and applied studies, which studied and evaluated different types of these models in the statistical analysis of spatial time series data were presented, This section discusses a set of those studies in a theoretical way.

The spatial time series models have been defined as: “the models that used for the study of linear relationships between the variables in two dimensions: time and space” [3, 25]. Elhorst [25] assumes that, in the space-time model, there are relationships between the data gotten from several locales under the acceptance and the systematic dependency to the proportional distance between the locales. So, the conditional average of variable *Z*_*i*_(*t*), can be modeled by *i* and a linear function of the past values of the subject variables that obtained from its neighbor locales.

### STARMA models

These models are called the STARMA models because the variable *Z*_*i*_(t) can be expressed as a spatial dependence function in the previous observations *Z*_*i* = 1,…,*N*_(t−1),…,*Z*_*i* = 1,…,*N*_(t−K) and the previous random error limits *ε*_*i* = 1,…*N*_(t−1),…,*ε*_*i* = 1,…*N*_(t−k) in addition to the current random error limit *ε*_*i* = 1,…*N*_(t). The order rank is determined by the number of previous observations. The limits of the previous random error and the spatial order of the (*k*^*th*^) autoregressive and moving average included in the model. If the current observation *z*_*i*_(t) relied on the number (p) of the previous observations, the number (q) of the previous random error limits, the current random error limit *ε*_*i*_(t), the numbers (*λ*_*k*_) of the previous observations in the site (*k*), the number (*m*_*k*_) of the previous random error limits in the site (*k*), then *z*_*i*_(t) is said to follow a Space-Time Autoregressive Moving Average (*p*, *q*, *λ*_*k*_, *m*_*k*_) and it can be formulate as follows:

$$Z_{i}(t)=\sum_{k=1}^{p}\sum_{l=0}^{\lambda_{k}}\varphi_{kl}L^{\left(l\right)}Z_{i}(t-k)+\varepsilon_{i}(t)-\sum_{k=1}^{q}\sum_{l=0}^{m_{k}}\theta_{kl}L^{\left(l\right)}\varepsilon_{i}(t-k)$$ (5)

where Z_i_(t) is the observation of the random variable at site (*i*)_*i* = 1,2,…,N_, and time (*t*)_*t* = 1,2,…,*K*_. While *p* is the autoregressive order, *q* is the moving average order, *λ*_*k*_ is the spatial order of the *k*^*th*^ autoregressive term, m_k_ is the spatial order of the k^th^ moving average term, φ_kl_ is the autoregressive parameter at temporal lag k and spatial lag *l*, θ_kl_ is the moving average parameter at temporal lag k and spatial lag l, L^(*l*)^ is the N×1 vector of weights for spatial, and *ε*_*i*_(t) is the random normally distributed error vector at time (*t*) with *E*[*ε*_*i*_(t)] = 0 and

$$E[\varepsilon_{i}(t)\varepsilon_{j}(t+s)]=\left\{ \begin{array}{c} \sigma^{2},i=j,s=0\\ 0,otherwise. \end{array}\right.$$

We refers to this model as STARMA ${(p}_{\lambda_{1},\lambda_{2},\ldots {.,\lambda }_{p},}\;q_{m_{1},m_{2},\ldots {.,m}_{q}})$. We express the same model in vector form as

$$Z(t)=\sum_{k=1}^{p}\sum_{l=0}^{\lambda_{k}}\varphi_{kl}W^{\left(l\right)}Z(t-k)-\sum_{k=1}^{q}\sum_{l=0}^{m_{k}}\theta_{kl}W^{\left(l\right)}\varepsilon (t-k)+\varepsilon (t),$$ (6)

with *E*[*ε*(t)] = 0 and $E[{\varepsilon (t)\varepsilon (t+s)}^{\prime }]=\left\{ \begin{array}{l} \sigma^{2}I_{NT},\;s=0\\ \;0,otherwise. \end{array}\right.$

### STAR model

Two special sub-classes of the STARMA model are considered in this study. When *q* = 0, only autoregressive terms remain, and this model is referred to as space-time autoregressive or STAR model. The STAR model is given as:

$$Z(t)=\sum_{k=1}^{p}\sum_{l=0}^{\lambda_{k}}\varphi_{kl}W^{\left(l\right)}Z(t-k)+\varepsilon (t)$$ (7)

We refer to this model as STAR ${(p}_{\lambda_{1},\lambda_{2},\ldots {.,\lambda }_{p}})$.

### STMA model

Models that contain no autoregressive terms (*p* = 0) are referred to as STMA models. STMA model is given as:

$$Z(t)=\sum_{k=1}^{q}\sum_{l=0}^{m_{k}}\theta_{kl}W^{\left(l\right)}\varepsilon (t-k)+\varepsilon (t)$$ (8)

We refer to this model as STMA ${(q}_{m_{1},m_{2},\ldots {.,m}_{q}}).$

### Spatial weight matrix

The specification of the form of weights $W_{ij}^{\left(f\right)}$ for various positive *i*’s is a matter left to the model builder who may choose weights to reflect the configuration of the physical properties of a system. For example, the county system. The $W_{ij}^{\left(f\right)}$ may be chosen to reflect the physical properties of the observed system such as the length of the common boundary between contiguous counties *i* and *j*, the distance between the centers of counties, natural barriers such as rivers or mountains, and even the ease of accessibility of county *i* to county *j*. This last factor includes such things like the number of roads between *i* and *j*, the amount of public transportation available that connects the two county, and even the flow rates upon these avenues.

### Parameters estimation

After identifying the order of the model from the STARMA model family, the next step is to estimate the parameters. This section deals with the estimation of the STARMA subclass family’s parameters. It should be noted that the method of estimating STARMA models are not the same for each subclass. This is because the STAR is in linear form, whereas the STMA and mixed STARMA are in nonlinear form.

### Maximum likelihood estimation

The best estimates of the *φ*_*kl*_ and *θ*_*kl*_ from many points of view are the maximum likelihood estimates (MLEs) [1]. This is because our basic model formulation has errors that are purely white noise. The distribution of *ε* is multivariate normal with mean zero and variance-covariance matrix, *σ*^2^*I*_*NT*_. We define its probability density function as follow:

$$f(\varepsilon |\Phi ,\Theta ,\sigma^{2})={\left(2\pi \right)}^{-\frac{TN}{2}}{\left|\sigma^{2}I_{NT}\right|}^{-\frac{1}{2}}exp(-\frac{1}{2\sigma^{2}}\varepsilon^{\prime }\varepsilon )={\left(2\pi \right)}^{-\frac{TN}{2}}{\left(\sigma^{2}\right)}^{-\frac{TN}{2}}exp\left(-\frac{S(\Phi ,\Theta )}{2\sigma^{2}}\right)$$ (9)

where $S(\Phi ,\Theta )=\varepsilon^{\prime }\varepsilon =\sum_{i=1}^{N}\sum_{t=1}^{T}\varepsilon_{i}{\left(t\right)}^{2}.$

Since the ***ε***(*t*) are the unobservable random errors and the *Z*(*t*) are the quantities actually observed, it is necessary to recursively calculate the *ε*(*t*) from the observed *Z*(*t*). The appropriate equation is given as

$$\varepsilon (t)=Z(t)+\sum_{k=1}^{p}\sum_{l=0}^{\lambda_{k}}\varphi_{kl}W^{\left(l\right)}Z(t-k)-\sum_{k=1}^{q}\sum_{l=0}^{m_{k}}\theta_{kl}W^{\left(l\right)}\varepsilon (t-k)\;for\;t=1,2,\ldots ,T$$ (10)

The conditional likelihood function of *Φ*, Θ, and ***σ***^2^ is given as

$$L(\Phi ,\Theta ,\sigma^{2}|Z)={\left(2\pi \right)}^{-\frac{TN}{2}}{{(\sigma }^{2})}^{-\frac{TN}{2}}exp\left(-\frac{S_{*}(\Phi ,\Theta )}{2\sigma^{2}}\right)$$ (11)

where S_*_(*Φ*, Θ) is the function of conditional sum of squares: $S_{*}(\Phi ,\Theta )={\hat{\varepsilon }}^{\prime }\hat{\varepsilon },$ where the $\hat{\varepsilon }$ vector is calculated via Eq (10) with Z(*t*) and *ε*(*t*) set equal to zero for *t*<1. The conditional MLEs of *Φ*, Θ, *σ*^2^ are given as ${\hat{\sigma }}^{2}={\left(NT\right)}^{-1}S_{*}(\hat{\Phi },\hat{\Theta })$, and $\hat{\Phi },\hat{\Theta }$ that minimize S_*_(*Φ*, Θ).

## COVID-19 in Makkah region

The data collected relates to the total reported confirmed cases of COVID-19 of three districts of Saudi Arabia, i.e., Makkah, Jeddah, and Taif of Makkah region. The dataset was obtained from 31 May to 11 October 2020 from the application programming interface (https://covid19.moh.gov.sa/). We employ ARIMA and STARIMA models utilizing the neighbor spatial weights matrices.

The objective is to show how to choose the appropriate model to fit our data. It is necessary to know whether or not there are spatial effects resulting from curfew, and determine the type of spatial interaction effects to consider for the data. To this end, we will employ the three-stage model building procedures proposed by Box and Jenkins in modeling daily confirmed patient’s data of the Covid-19 pandemic for both ARIMA and STARIMA.

### Descriptive statistics of COVID-19 time series

Descriptive statistics of the prevalent confirmed cases of COVID-19 from 31 May to 11 October 2020 are given in Table 1. That is, the prevalence of COVID-19 in Jeddah, Makkah, and Taif cases as reported during this period.

**Table 1:** Descriptive statistics of COVID-19 cases.

Location	Minimum	Maximum	Mean	Std. Deviation	Skewness	Kurtosis
Jeddah	3	577	140.903	139.561	1.285	3.711
Makkah	8	623	132.828	110.63	1.494	5.140
Taif	0	306	68.776	77.469	1.234	3.539

### Implementation

**Fig 1:**
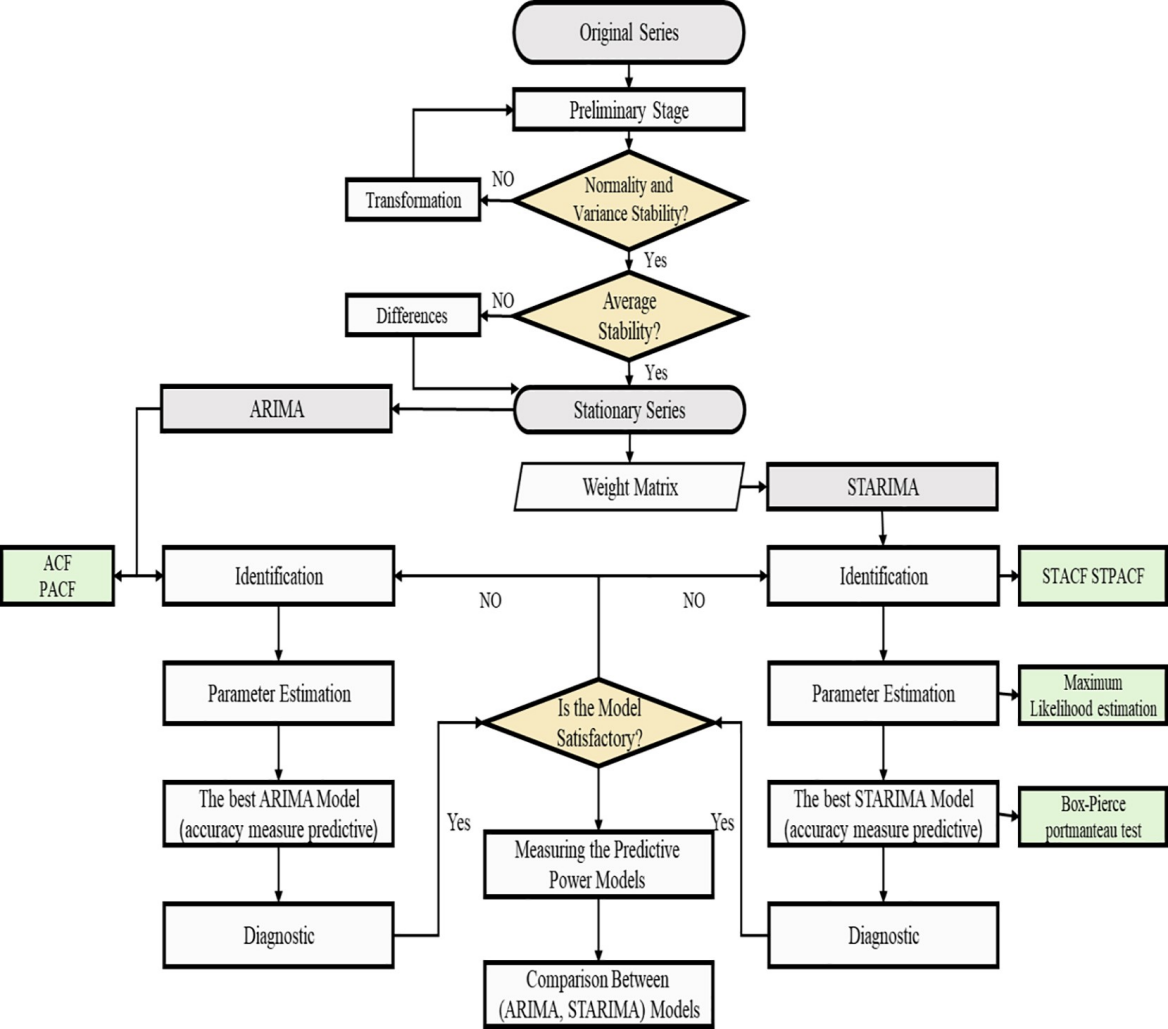
Applied study steps for ARIMA and STARIMA model.

Models were implemented using R (Version 3.6). Fig 1 shows a flow chart for the implementation of STARIMA and ARIMA models in our analysis.

### Preliminary stage

**Fig 2:**
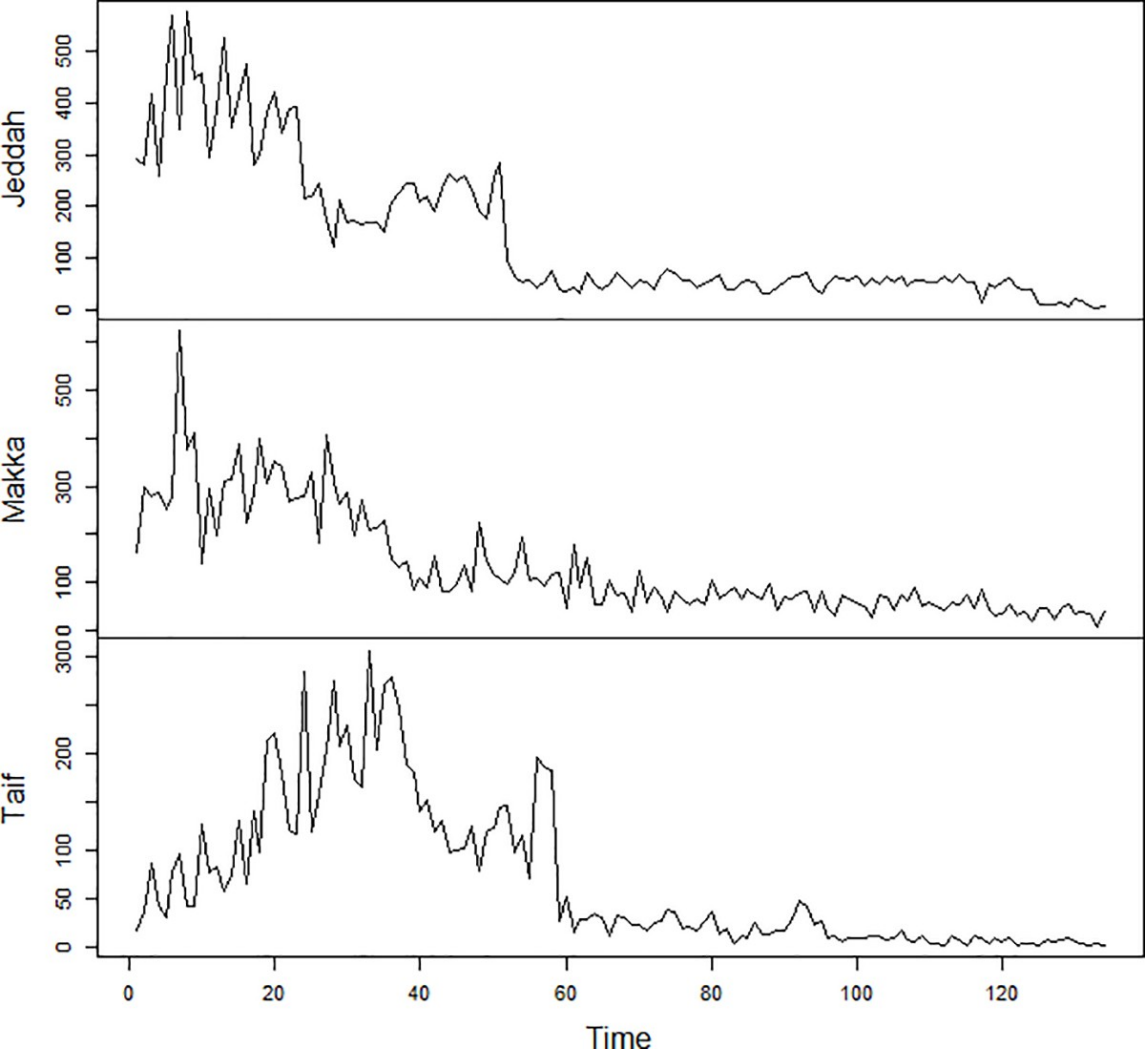
Time series plot of daily confirmed cases of COVID-19.

**Fig 3:**
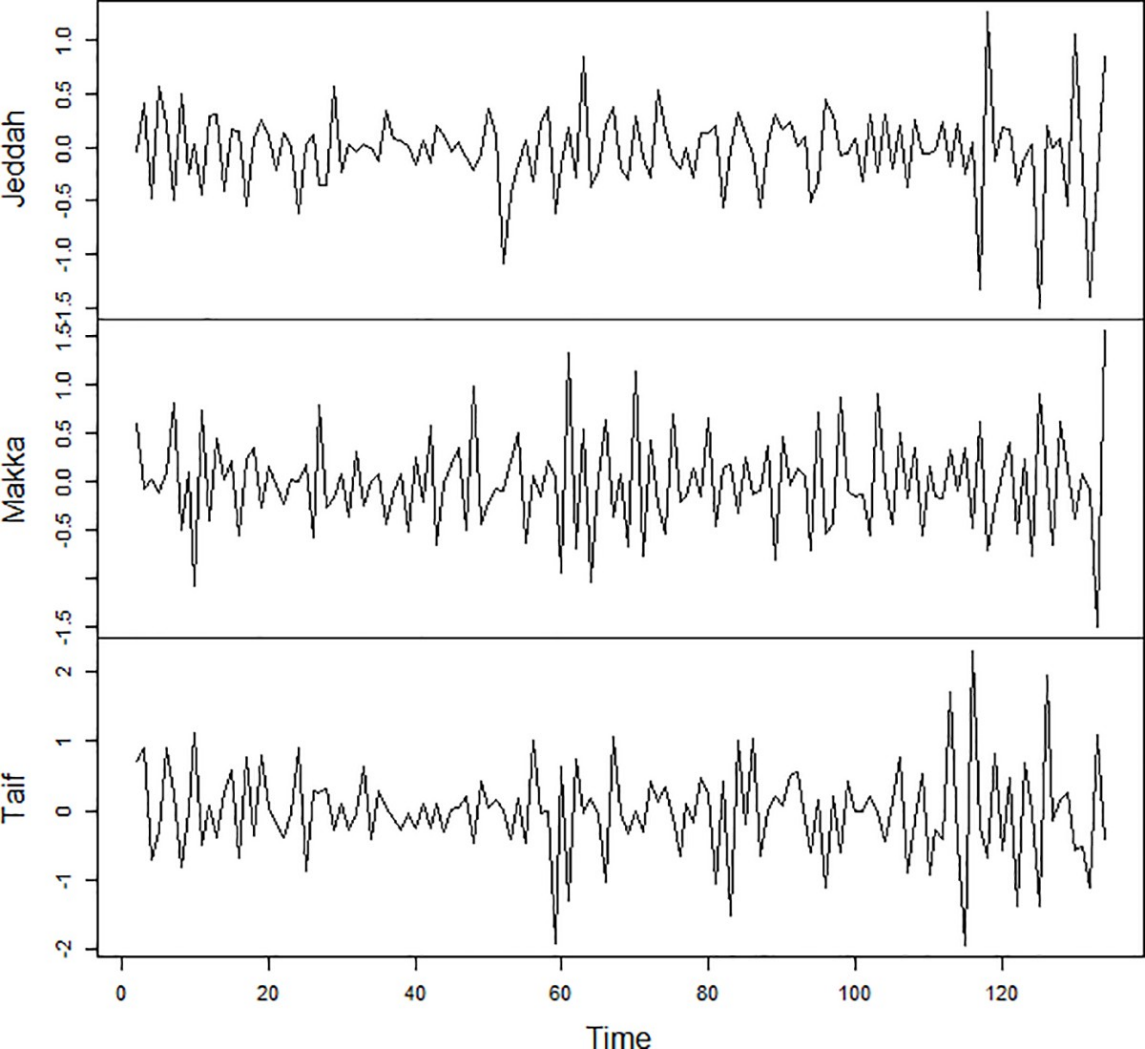
Time series plot of the differenced daily confirmed cases of COVID-19.

The preliminary analysis of the data was done by using time plots of the series as shown in Fig 2. A visual inspection of the time series plot indicates that the confirmed cases of COVID-19 in three districts of Saudi Arabia are not stationary series. So, the data must be transformed to achieve stationary. The series was transformed by taking the first difference of the natural logarithm of the values of each series to obtain stationary, the equation representing the transformation is given by: *W*_*t*_ = *ln* (*X*_*t*_)−*ln* (*X*_*t*−1_). After the transformation, Fig 3 shows that *W*_*t*_ series is stationary and have no trend. The value of d = 1 in ARIMA and STARIMA models.

### ARIMA models for COVID-19 in Makkah region

The univariate ARIMA model is fitted for the three districts separately, according to Box-Jenkins ARIMA approach.

#### Identification

**Fig 4:**
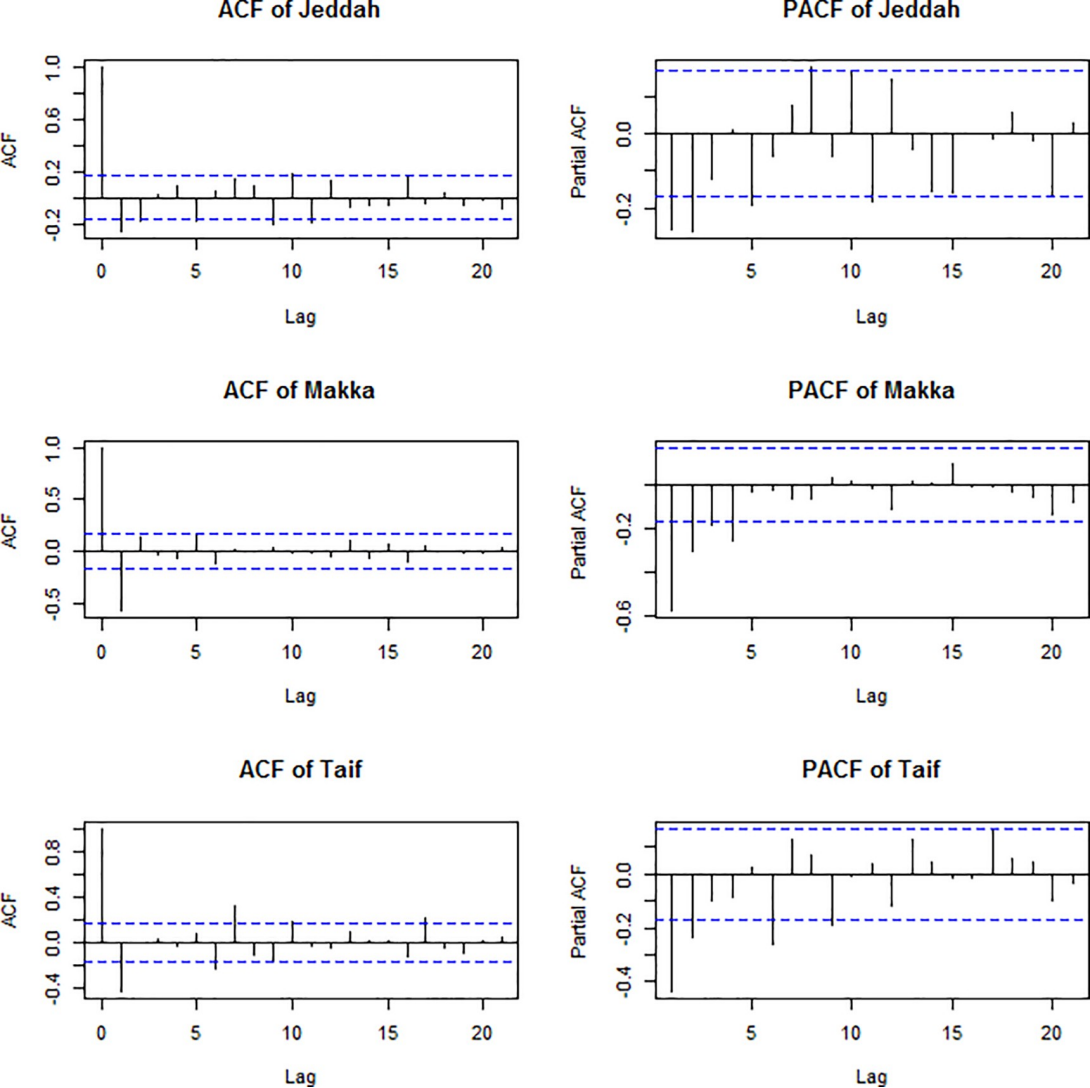
ACF and PACF of the differenced series.

To identify the value of the two parameters p and q from ACF and PACF of the differenced series and compare the two plots shown in Fig 4. The ACF of all series has a significant spike at time lag (1), described by the first order of moving average. Note that the time lag (0) was ignored. The PACF of the three series have a significant spike at time lag (1) and lag (2), which is described by the order of the autoregressive process.

#### Parameters estimation

Base on the ACF and PACF from the first stage and "auto.arima" function in R package "forecast," the Box-Jenkins ARIMA model has been fitted for the three locations separately. As a result, it was the best models as shown in Table 2. The autoregressive and moving average parameters of our models for the three districts (Jeddah, Makkah, and Taif). After the candidate model selection and parameters estimation by the maximum likelihood estimation method. Furthermore, we can note that all moving average parameters of three models are statistically significant at 0.05.

**Table 2:** The results of ARIMA model.

Location	Model	Statistic	AR Parameter	MA Parameter	Intercept
Jeddah	ARIMA (0,1,1)	Estimate		-0.4112***	
		SE		0.0892	
		t.value		-4.6088	
		p.value		0.0001	
Makkah	ARIMA (1,1,1)	Estimate	-0.2172	-0.7431***	-0.0177**
		SE	0.1112	0.0858	0.0067
		t.value	-1.9529	-8.6646	-2.6330
		p.value	0.0530	0.0001	0.0095
Taif	ARIMA (0,1,1)	Estimate		-0.6009***	
		SE		0.0748	
		t.value		-8.0354	
		p.value		0.0001	

Note

**, *** indicate statistical significance at the 0.05, 0.001, respectively.

#### Diagnostics

**Fig 5:**
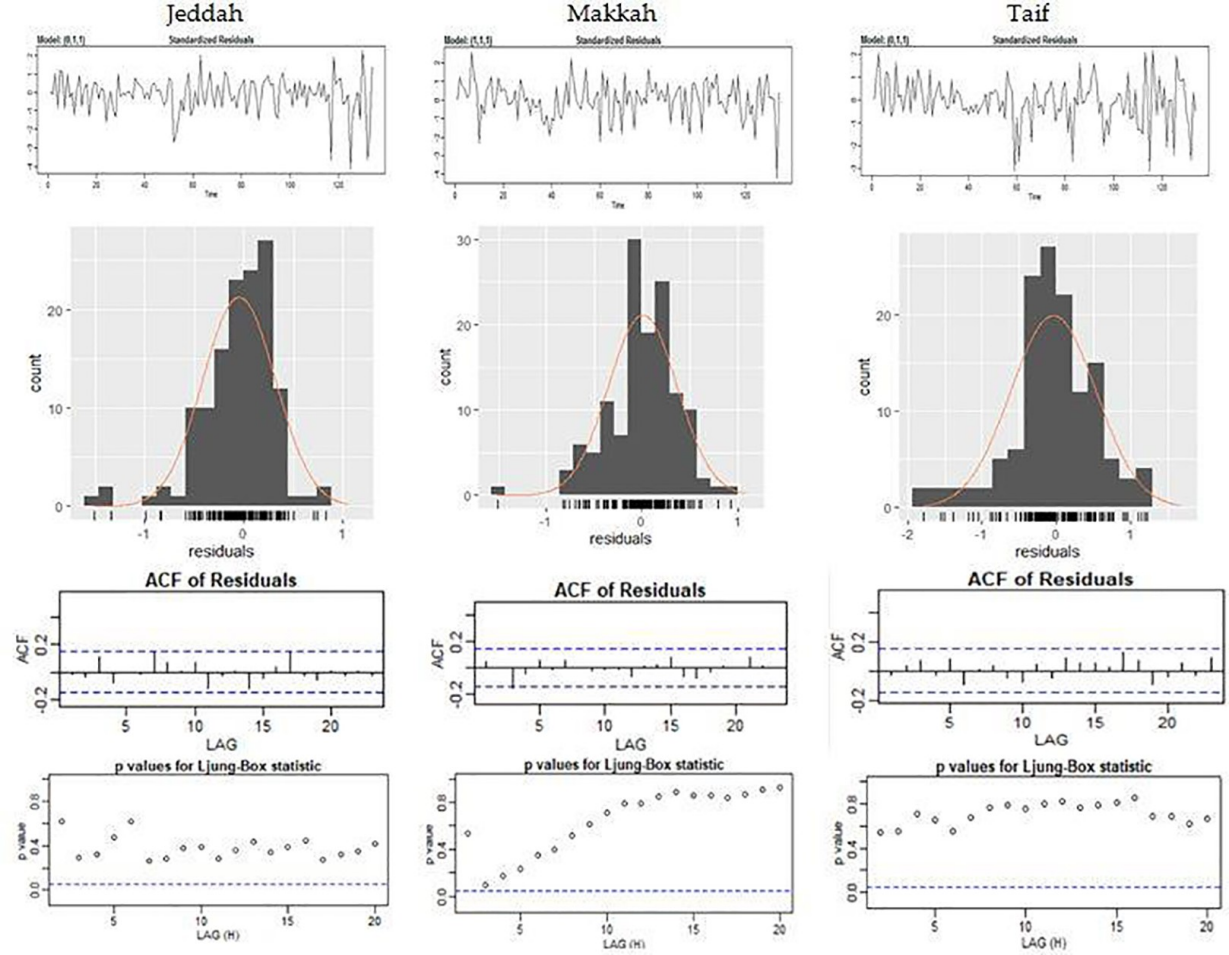
Residuals analysis plots of the three ARIMA model.

According to Box-Jenkins approach, diagnostic testing of a model involves checking the normality and the stationary of the residuals [26]. Fig 5 shows that the residuals are distributed normally. From the autocorrelation function of the residual, it is clear that all the autocorrelation coefficients are between the two limits of the confidence interval. This indicates the independence of the residuals and the satisfaction of the assumption of independence.

### STARIMA models

In this stage, we consider the multivariate STARIMA model for the three districts together.

#### Construction spatial weight matrix

For the STARIMA model, a spatial weight matrix was created. First order spatial neighborhood spatial matrices in Table 3 were gotten agreeing to the spatial neighborhood relationship of the three counties in Makkah region. We assumed the diagonal elements of the first-order contiguity matrix equal zero. The spatial weight matrix was also constructed by assigning equal weight to each neighbor.

**Table 3:** Spatial weight matrix of order and one.

Spatial weight matrix of order zero			
Location	Jeddah	Makkah	Taif
Jeddah	1	0	0
Makkah	0	1	0
Taif	0	0	1
First order spatial weight matrix			
Location	Jeddah	Makkah	Taif
Jeddah	0	1	0
Makkah	0.5	0	0.5
Taif	0	1	0

### STARMA model fitting

In this article, we estimated the STARMA model using the three-stage procedure according to [1]. As discussed in methodology section, STARMA estimation procedure is an extension of the Box-Jenkins methodology in spatio-temporal set up. As in ARIMA, it has three stages of model building: the model identification, estimation, and diagnostic checking.

#### Identification

**Fig 6:**
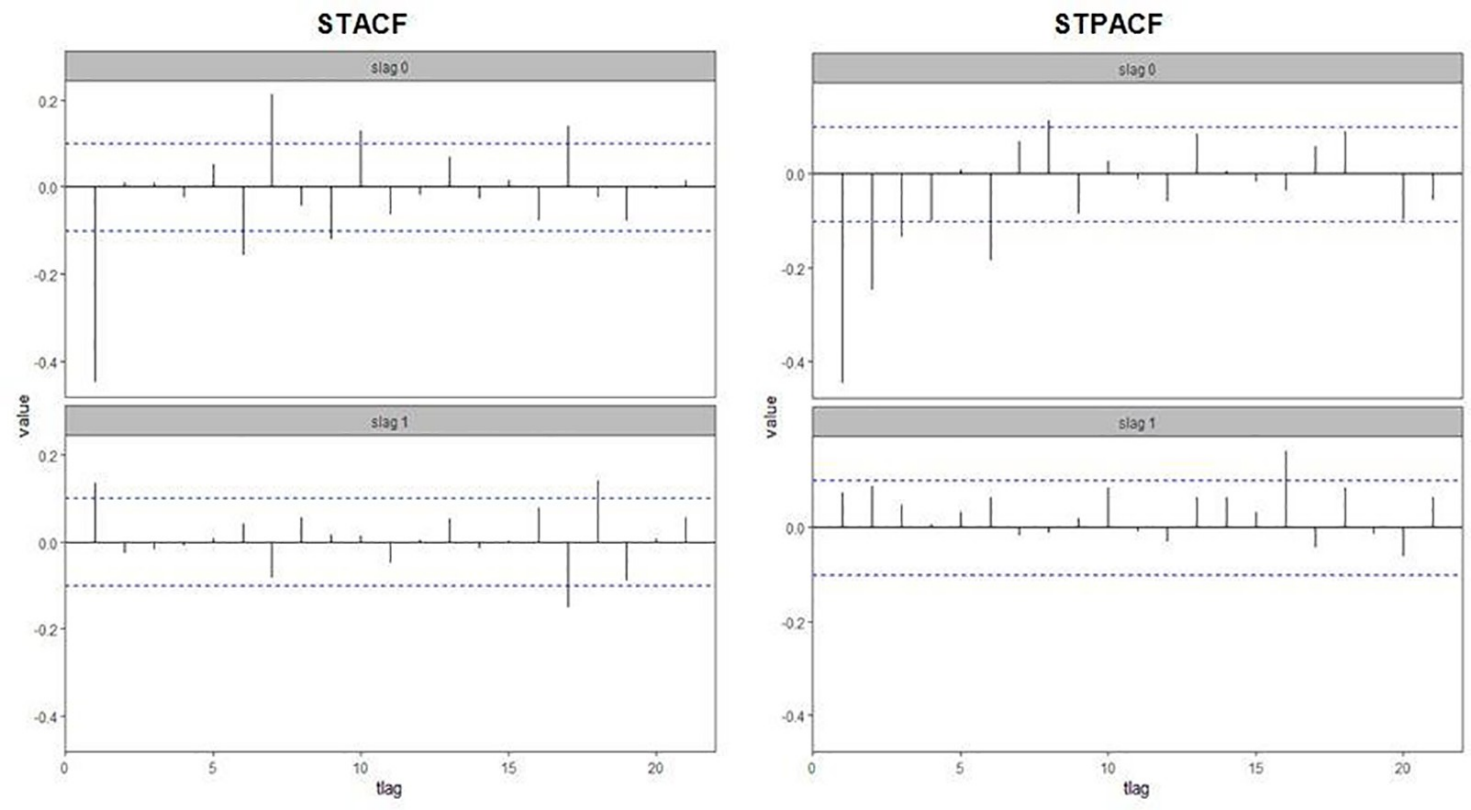
STACF and STPACF of the differenced series.

Model identification is the most vital stride to utilize the forms of spatio-temporal models: STAR, STAMA, and STARMA. To identify the value of the parameters, we used STACF, and STPACF of the differenced series for the three locations (see Fig 6). According to Fig 6, we can conclude that the appropriate model for the data is one of the following: STARIMA (0,1,1_0_), STARIMA (0,1,1_1_), or STARIMA (1_0_,1,1_1_).

#### Parameters estimation

The estimated parameters of the identified models (STARIMA (0,1,1_0_)_,_ STARIMA (0,1,1_1_) and STARIMA (1_0_,1,1_1_)), using the maximum likelihood method, given in Table 4, alongside their standard errors, t.values, and p.values.

**Table 4:** The results of the estimated STARIMA models.

Spatial Order	Model	Statistic	Spatial	Spatial	
			AR Parameters	MA Parameters	
			*φ*_10_	*θ*_10_	*θ*_11_
Lag(0)	STARIMA (0,1,1_0_)	Estimate		-0.5882***	
		SE		0.0508	
		t.value		-11.5840	
		p.value		< 2.2e-16	
Lag(1)	STARIMA (0,1,1_1_)	Estimate		-0.5807***	0.1976**
		SE		0.05132	0.0611
		t.value		-11.3159	3.2334
		p.value		< 2.2e-16	0.0013
	STARIMA (1_0_,1,1_1_)	Estimate	-0.0991	-0.48063***	0.1967**
		SE	0.0866	0.1013	0.0627
		t.value	-1.1443	-4.7431	3.1353
		p.value	0.2532	2.942e-06	0.0018

Note

**, *** indicate statistical significance at the 0.05, 0.001, respectively.

#### Choosing the best spatial model

To choose the best spatial model, we depended on the following accuracy measures: Mean Absolute Error (MAE), Mean Squared Error (MSE), and Root Mean Squared Error (RMSE), to compare the forecasting performances of the STARIMA models. According to the results in Table 5, the lowest MAE, MSE, and RMSE values is STARIMA (1_0_,1,1_1_), so the best spatial model is STARIMA (1_0_,1,1_1_) for the dataset.

**Table 5:** Accuracy measures of STARIMA models.

Location	Measure	STARIMA (0,1,1)_0_	STARIMA (0,1,1_1_)	STARIMA (1_0_,1,1_1_)
**Jeddah**	**MAE**	0.1668	0.1710	0.1560
	**MSE**	0.0516	0.0544	0.0487
	**RMSE**	0.2273	0.2333	0.2206
**Makkah**	**MAE**	0.1800	0.1775	0.1706
	**MSE**	0.0557	0.0538	0.0507
	**RMSE**	0.2360	0.2319	0.2252
**Taif**	**MAE**	0.2489	0.2493	0.2482
	**MSE**	0.1167	0.1156	0.1150
	**RMSE**	0.3416	0.3400	0.3391

#### Diagnostics

**Fig 7:**
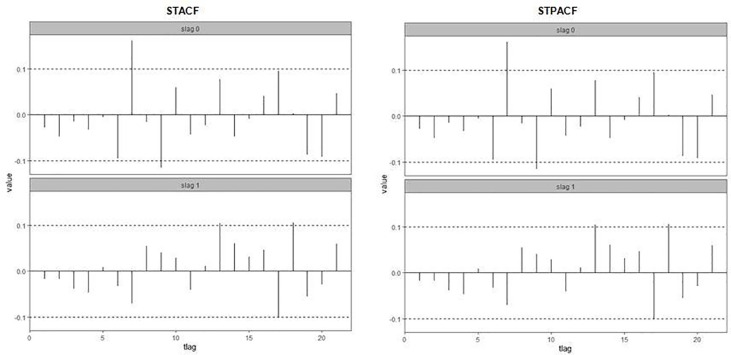
STACF and STPACF of STARIMA (1_0_,1,1_1_) residuals.

The diagnostic checking stage involves checking the independence of the residuals of STARIMA (1_0_,1,1_1_) model. We applied the multivariate Box-Pierce non-correlation test (Chi-square = 51.89, df = 42, p.value = 0.141), since the p.value of Box-Pierce test more than 0.05, then we can’t reject non-correlation hypothesis, this means that the residuals were uncorrelated (independent). Furthermore, from the STACF and STPACF functions of the residual (see Fig 7), it was clear that the independence of the residuals and the satisfaction of the independence assumption.

### Measuring the predictive power of models

MAE, MSE, and RMSE measures were computed to compare the forecasting performances of the best traditional ARIMA model and the best STARIMA models under considerations for all the locations separately after the curfew. According to the results in Table 6, the lowest MAE, MSE, and RMSE values of the proposed STARIMA model for Jeddah, Makkah, and Taif after the curfew, it is confirmed that the interpretation of the curfew STARIMA model performed better than the Box-Jenkins ARIMA model in the locations for training dataset.

**Table 6:** Accuracy measures of predictive power for ARIMA and STARIMA models.

Location	Models	MAE	MSE	RMSE
**Jeddah**	ARIMA (0,1,1)	0.2619	0.1358	0.3685
	STARMA (1_0_,1,1_1_)	0.1560	0.0487	0.2206
**Makkah**	ARIMA (1,1,1)	0.2712	0.1285	0.3585
	STARMA (1_0_,1,1_1_)	0.1706	0.0507	0.2252
**Taif**	ARIMA (0,1,1)	0.4247	0.3303	0.5747
	STARMA (1_0_,1,1_1_)	0.2482	0.1150	0.3391

## Conclusion

Box-Jenkins univariate ARIMA models are most popularly used in univariate time series data, whereas their applications are limited when it comes to multivariate spatio-temporal time series data. Unlike the univariate ARIMA models, STARIMA models have a smaller number of parameters. For the illustrated dataset, the STARIMA model has only three parameters for the three locations, whereas ARIMA model has numerous parameters, under such cases over parameterization may prompt to lower sum of squares of residuals. In conclusion, we studied the trend pattern of the COVID-19 outbreak in Makkah, Jeddah, and Taif in Saudi Arabia. We found out that the best prediction model for forecasting the trend of daily confirmed cases in Makkah, Jeddah, and Taif is STARIMA (1_0_,1,1_1_).

Based on the results obtained, one can infer that the STARIMA model outperforms the univariate ARIMA model. The outperformance of the STARIMA model over the univariate ARIMA model could be due to the inclusion of spatial information i.e. neighboring effect in the form of spatial weight matrix. Accordingly, we recommend using spatial analysis for predicting the COVID-19 confirmation cases and limiting the increase of these cases by taking appropriate preventive measures such as social distancing, curfew, banning gatherings, etc.

As future work for our study, we can use the updated spatial-temporal models, such as Bayesian spatial-temporal models [13–16] or spatial fixed/random effects panel models [27–29].
